# Effects of Konjaku Flour on the Gut Microbiota of Obese Patients

**DOI:** 10.3389/fcimb.2022.771748

**Published:** 2022-03-01

**Authors:** Yu Li, Yongbo Kang, Yuhui Du, Minghui Chen, Liqiong Guo, Xinwei Huang, Tingting Li, Shi Chen, Fan Yang, Fubing Yu, Jingan Hong, Xiangyang Kong

**Affiliations:** ^1^ Medical Faculty, Kunming University of Science and Technology, Kunming, China; ^2^ School of Medicine, Southern University of Science and Technology, Shenzhen, China; ^3^ School of Basic Medical Sciences, Shanxi Medical University, Taiyuan, China; ^4^ Nutrition Department, The First People’s Hospital of Yunnan Province, Kunming, China; ^5^ Department of Gastroenterology, The Second People’s Hospital of Yunnan Province, Kunming, China

**Keywords:** obesity, gut microbiota, konjaku flour, obesity index, blood parameters

## Abstract

**Objective:**

Gut microbiota have been thought to play a role in the emergence of obesity and metabolic disorders, thus dietary fiber may be an effective strategy for the management of obesity by modulating the gut microbiota. The aim of the present study was to investigate the effects of konjaku flour (KF) supplementation on treating obesity and regulating intestinal microbiota in obese adults.

**Methods:**

In a 5-week, randomized, double-blind, place-controlled trial, sixty-nine obese volunteers aged 25 to 35 with body mass index ≥28 kg/m^2^ were randomly assigned to receive KF or placebo (lotus root starch). Obesity index, blood parameters, and gut microbiota were analyzed.

**Results:**

KF remarkably reduced the body mass index (BMI), fat mass, percentage body fat (PBF), serum triglyceride (TG), glycated hemoglobin A1c (HbA1c), aspartate aminotransferase (AST), and alanine aminotransferase (ALT) levels in the patients (*p <*0.05 or *p <*0.01). Meanwhile, high-throughput sequencing and bioinformatics analysis showed that the konjac flour treatment notably increased the α-diversity and changed the β-diversity of intestinal microflora in patients (*p <*0.01). Moreover, konjac flour could also evidently increase the abundance of some of the beneficial microorganisms related to obesity of patients, such as *Lachnospiraceae*, *Roseburia*, *Solobacterium*, *R. inulinivorans*, *Clostridium perfringens*, and *Intestinimonas butyriciproducens*, and reduce the abundance of the harmful microorganisms, such as *Lactococcus*, *Bacteroides fragilis*, *Lactococcus garvieae*, *B. coprophilus*, *B. ovatus*, and *B. thetaiotaomicron* (*p <*0.01). Specifically, *C. perfringens* was significantly negatively correlated with serum total cholesterol (TC) (*p <*0.01).

**Conclusion:**

These results suggested that KF can achieve positive effects on treating obesity, which manifest on reducing BMI, fat mass, blood glucose, and blood lipid, improving hepatic function, and also regulating intestinal microfloral structure. Therefore, changes in gut microbiota may explain in part the effects of KF.

## Introduction

Obesity can be said to be one of the biggest health care challenges in the industrialized world. In the past few decades, the prevalence of obesity has been increasing worldwide (Mullin and Delzenne, 2014). Currently, there are more than 1 billion overweight or obese adults in the world (Bouchard, 2000). A growing epidemic is threatening all countries. Therefore, the American Medical Association (AMA) declared obesity as a disease in June 2013 (Funk et al., 2016). Treating the huge burden of obesity-related diseases to society urgently requires effective methods to manage this public health problem (Schwiertz et al., 2010; Chang et al., 2015).

The gut microbiota may be regarded as an “organ” that contributes to metabolism and plays a role in energy storage. The human gut microbiota is composed of trillions of bacteria. Recent studies on animal models and human subjects have shown that there are differences in the composition of the gut microbiota associated with obesity (Ley et al., 2005). In addition, the evidence so far shows that the intestinal microbiota affects systemic metabolism by affecting energy balance, inflammation and intestinal barrier function, integrating peripheral and central food intake regulatory signals, thereby changing body weight (Kang and Cai, 2017). At the same time, these data indicate that the types of intestinal commensal bacteria may play a pathogenic or protective role in the development of obesity (Kang and Cai, 2017). The evidence so far suggests that manipulation of the gut microbiome may represent an effective treatment method for preventing or managing obesity, so dietary fiber may be an effective strategy for improving and managing obesity by regulating the gut microbiota (Kang and Cai, 2017). Diet has an impact on the composition of the gut microbiota (Hildebrandt et al., 2009; Jumpertz et al., 2011; Wu et al., 2015), and these bacteria have been proposed to participate in the development of obesity and diabetes (Ley et al., 2006; Brugman et al., 2006; Cb et al., 2010).

Konjac flour (KF) mainly contains konjac glucomannan (KGM), which is a water-soluble polysaccharide (dietary fiber), made from the tuber of the Araceae perennial plant Konjac K. Koch (Zhang et al., 2005). For centuries, it has been cultivated in Asian countries as a food source and a traditional Chinese medicine ingredient. Konjac products are listed as one of the “Top Ten Health Foods” by the World Health Organization (Chua et al., 2010; Tester et al., 2012; Li et al., 2013). The important health benefits of KF include lowering cholesterol (Davé and Mccarthy, 1997), normalizing the concentration of triglycerides (TG) in the blood (Lee and Dugoua, 2011), improving blood sugar levels (Martino et al., 2013), immune function (Chen et al., 2003), and promoting intestinal activity (Chiu and Stewart, 2012), and wound dressing (Onitake et al., 2015). KF is considered to be a non-digestible dietary fiber, which can resist hydrolysis through the action of digestive enzymes in the human intestine (Huang et al., 2015). Therefore, KF is of great significance for maintaining the homeostasis of the intestinal flora and protecting the intestinal barrier, so it is often referred to as a prebiotic. However, to date, there is little clinical information on the effects of KF on body weight, obesity-related diseases, and gut microbiota after dietary KF supplementation.

The purpose of this study was to examine the clinical efficacy of KF in obese adults. The obesity index, blood parameters and gut microbiota were analyzed.

## Materials and Methods

### Study Population

Participants in the clinical trial were recruited from December 2015 to April 2017 in the Nutrition Department of Yunnan First People’s Hospital in Kunming, China. Considering the influence of daily diet on gut microbiota, we make sure participants had relatively similar diet structure by questionnaires that included: (1) the number of midnight snack: hardly eat, 1–2 times/week, 3–4 times/week, or almost eat every day; (2) type of night snack: fruit, fried food, sweet cakes, dairy products, starch food, fried food and starch food, or cakes and fruit; (3) control the type of diet: controlling staple food quantity, increasing vegetable quantity, prohibiting sweet cakes and sugary drinks, prohibiting fat meat and animal viscera, prohibiting fried food, not controlling diet, or controlling staple food quantity, fat meat and animal viscera; (4) Beverage type: none, carbonated beverage, juice, milk tea and coffee tea, or tea; (5) Weekly alcohol intake. Of the 90 registered obese adults whose diet structure is roughly as follows: (1) the number of midnight snack: 1–2 times/week; (2) type of night snack: fried food; (3) control the type of diet: controlling staple food quantity; (4) Beverage type: carbonated beverage; (5) almost no alcohol consumption.) screened, 69 were included. According to a simple randomization procedure (computer random number), the participants were randomly assigned to one of the two treatment groups. The subjects included men and non-pregnant women aged between 25 and 35 years with a body mass index (BMI) ≥28 kg/m^2^.

Subjects are excluded if they are taking drugs that may affect weight changes, namely, antidiabetic drugs, lipid-lowering drugs, or antifungal drugs, or if they have endocrine, cardiovascular, thyroid, or chronic liver disease. If they underwent surgery to lose weight, took probiotics or antibiotics within one month, or if their weight changed more than 5% within three months, then subjects were also excluded.

### Study Design

**Figure 1:**
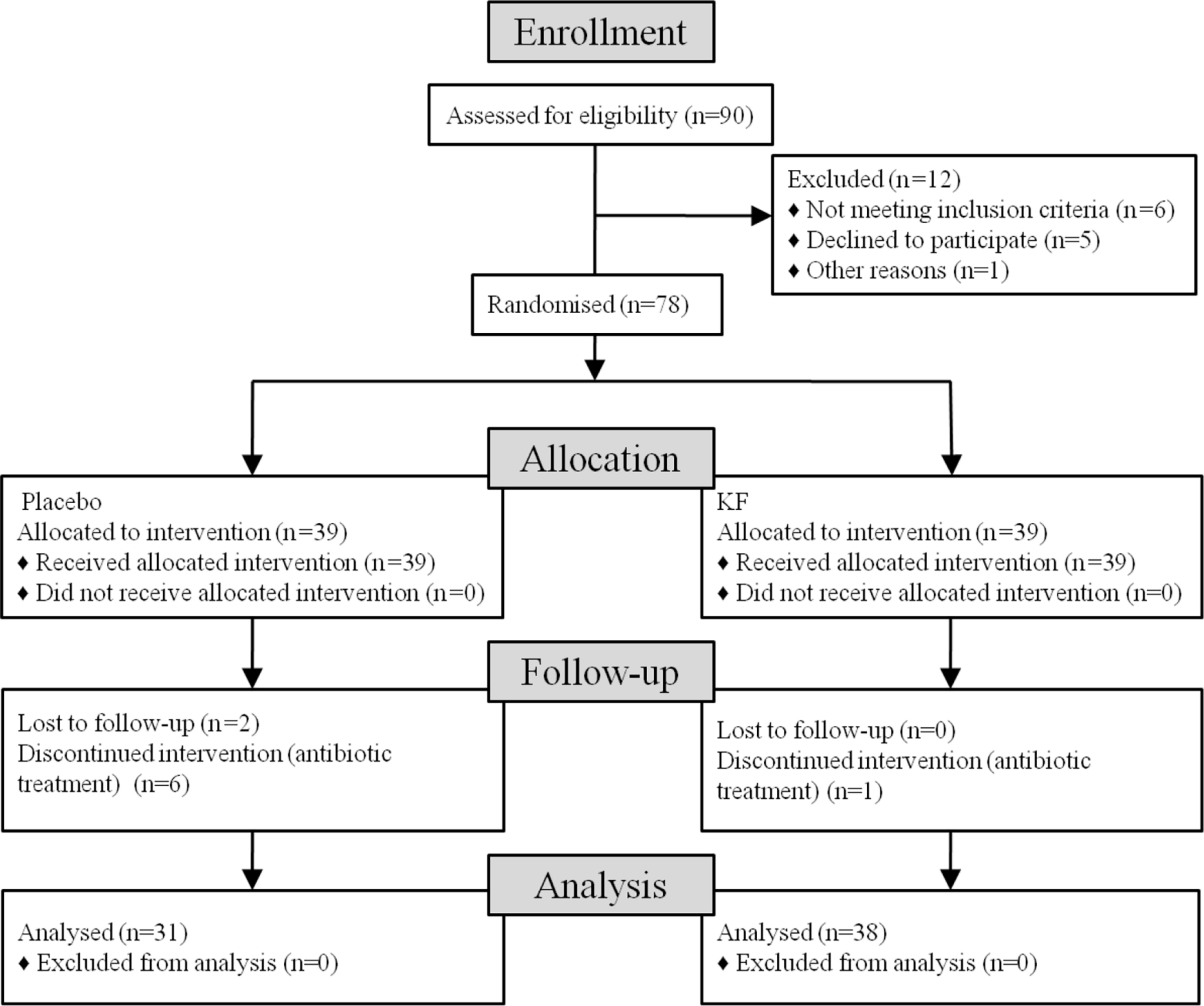
Flowchart shows the recruitment and retention of patients in the study.

This is a 5-week double-blind, placebo-controlled trial. The subjects were randomly assigned to one of the two groups and received 10 g of lotus root starch placebo or KF per day (5 g lotus root starch placebo or 150 ml of warm water plus KF 15 min before breakfast and dinner) (**Figure 1**). By emphasizing the intervention personnel and participants, blindness and balance are strictly maintained. Also, all investigators, staff, and participants were unaware of the outcome measurement and test results. This study was approved by the Ethics Committee of Kunming University of Science and Technology School of Medicine. All volunteers provided written informed consent before the study.

### Laboratory Analysis

Height was measured at the beginning of the study, and weight and waist circumference were measured at weeks 0 and 5. At baseline and week 5, the subjects received a DXA scan (Hologic QDR 4500, Hologic, Inc., Bedford, MA, USA) to measure fat mass and body fat percentage. Blood samples were collected at recruitment (week 0) and at the end of the trial (week 5). Serum glucose, HbA1c, serum total cholesterol (TC), low-density lipoprotein cholesterol (LDL-C), high-density lipoprotein cholesterol (HDL-C), triglycerides (TG) and liver function indicators (aspartate aminotransferase) (AST) and an automatic biochemical analyzer was used to measure alanine aminotransferase (ALT). Stool samples were collected at recruitment (week 0) and at the end of the trial (week 5). After collecting the stool sample, it was transported to the laboratory in an ice box and stored at −80°C for microbiota analysis.

### Fecal DNA Extraction and Illumina Miseq Sequencing

To analyze the microbial content, the QIAamp DNA Stool Mini Kit (Qiagen, USA) was used to extract total genomic DNA from stool samples according to the manufacturer’s instructions. Forward primer F-5’CCTACGGGRSGCAGCAG3’ and reverse primer R-5’GGACTACVVGGGTATCTAATC3’ were used to amplify the bacterial 16S rRNA gene V3–V4 region. The PCR method and DNA purification were performed according to the Illumina Miseq 16S metagenomic sequencing library preparation protocol. For the first round of PCR, the PCR mix (25 µl) contained 12.5 µl 2× KAPA HiFiHotStartReadyMix (KAPA Biosystem, Woburn, MA, USA), 2.5 µl microbial genomic DNA (5 ng/µl) and 5 µl amplicon for PCR, respectively with Forward (1 μM) and reverse (1 μM) primers. The PCR protocol followed was: 95°C initial denaturation for 3 min, then 25 cycles of amplification, 95°C denaturation for 30 s, 55°C annealing for 30 s, 72°C extension for 30 s, and finally 72°C extension for 5 min. Gel electrophoresis was used to observe the PCR product to confirm the size of the amplicon (630 bp). In the second round, the PCR products were purified using AMPure XP beads. Index PCR used Nextera XT Index Kit to connect dual index and Illumina sequencing adapters. Index PCR was performed on all purified DNA using MJ Research PTC-200P thermal cycler (MJ Research, Waltham, USA). PCR mix (50 μl) contained 25 μl 2× KAPA HiFiHotStartReadyMix, 5 μl Nextera XT Index 1 Primers (N7XX) (1 μM), 5 μl Nextera XT Index 2 Primers (S5XX) (1 μM), 10 μl PCR pure water, and 5 μl purified DNA. PCR conditions are: initial denaturation at 95°C for 3 min, then denaturation at 95°C for 30 s, annealing at 55°C for 30 s, extension at 72°C for 30 s, 8 cycles of amplification, and finally extension at 72°C for 5 min. Gel electrophoresis was used to visualize the indexed PCR amplicons to confirm the amplification of the appropriate size product (630 bp). Then AMPure XP beads were used to purify the indexed PCR products. The purified index PCR product was quantified on a NanoDrop ND2000 spectrophotometer (Thermo Scientific, Wilmington, DE), diluted to a working concentration of 20 ng/μl with 10 mM Tris pH 8.5, and combined equimolar amounts (100 ng). After separating the products by electrophoresis on a 2% agarose gel, a Zymo clean Gel DNA Recovery Kit (Zymo Research Corp., Irvine, CA, USA) was used to recover and concentrate the PCR products of the correct size. The final combined DNA concentration was measured by using the Invitrogen Qubit platform (52.4 ng/μl).

The large number of 16S rRNA gene reads generated by the Illumina Miseq sequencer were initially quality trimmed using Illumina standard software tools. The 16S rRNA readings were processed according to the previously described QIIME pipeline (Caporaso et al., 2010). In short, 97% homology with USEARCH and NCBI’s 16SMicrobial dataset (https://github.com/mtruglio/QIIME_utilities) and taxonomy were used to cluster sequences into operational taxa (OTU).

### Bioinformatics and Statistics Analysis

The intestinal microbial analysis pipeline was based on Qiime1 (http://qiime.org/) and R (https://www.r-project.org/). The data are expressed as the mean ± SD. For cross-sectional analyses of baseline characteristics and comparison of the relative abundance of each taxonomic group, differences were indicated using the Paired two-tailed Student’s t-test. *P*-value less than 0.05 was considered as statistical significance.

Based on the RDP classifier version 2.2 algorithm, the Greengene database was used to annotate OTU classification information. The OTU abundance data are normalized using the standard sequence number corresponding to the sample with the least sequence. Check the relative proportions of each OTU at the phylum, class, order, family, genus, and species level. QIIME versions 1.7.0 and R 3.4.1 were used to analyze the diversity of Alpha (within the community) and beta (between the communities). For beta diversity, unweighted (considering the presence or absence of each species) UniFrac was used to generate principal coordinate analysis (PCoA) plots. The unweighted paired group method that used arithmetic mean (UPGMA) clustering was used as a hierarchical clustering method to explain the distance matrix using average links. A Spearman correlation analysis was performed to determine the correlation. A *p*-value of ≤0.05 is considered statistically significant.

## Results

### Effects of KF Treatment on Obesity Index and Blood Parameters

Ninety subjects began the study. A total of 38 KF subjects and 31 control subjects completed the study for a total study retention rate of 76.7%. Before the intervention, there were no significant differences in the obesity index and blood parameters between the KF and placebo groups (**Table 1**). The consumption of KF and placebo did not cause any adverse effects (**Table 1**). Body mass index (BMI), fat mass, percentage body fat (PBF), triglyceride (TG), glycated hemoglobin A1c (HbA1c), aspartate aminotransferase (AST), and alanine aminotransferase (ALT) were significantly reduced in KF consumers (**Table 1**) (*p <*0.05 or *p <*0.01). There were no significant differences in the control group (**Table 1**).

**Table 1:** Obesity index and blood parameters in obese patients at the baseline and/or after placebo and KF treatment.

Characteristics	Placebo group (n = 31)			Konjac flour group (n = 38)		
	Baseline	After treatment	*p*-value	Baseline	After treatment	*p*-value
Age (years)	30.60 ± 5.72			31.14 ± 7.05		
BMI (kg/m^2^)	31.22 ± 5.13	31.48 ± 5.21	0.347	31.41 ± 4.90	28.98 ± 4.80	<0.001
Waist/hip ratio	0.93 ± 0.06	0.92 ± 0.06	0.102	0.93 ± 0.05	0.92 ± 0.05	0.453
Fat mass (kg)	31.18 ± 12.24	31.83 ± 11.95	0.603	31.32 ± 10.42	29.02 ± 10.23	<0.001
PBF (%)	37.48 ± 6.79	38.11 ± 7.00	0.869	36.46 ± 7.13	34.58 ± 7.54	0.013
FBG (mmol/L)	5.28 ± 0.69	5.30 ± 0.53	0.130	5.20 ± 0.94	5.22 ± 0.71	0.822
HbA1c (%)	5.87 ± 1.00	5.99 ± 0.91	0.115	5.71 ± 0.84	5.54 ± 0.75	0.029
TC (mmol/L)	4.65 ± 0.65	4.60 ± 0.66	0.758	4.39 ± 0.95	4.30 ± 0.75	0.375
TG (mmol/L)	2.01 ± 0.59	1.95 ± 0.61	0.110	2.17 ± 1.64	1.78 ± 1.41	0.025
HDL (mmol/L)	1.11 ± 0.11	1.05 ± 0.19	0.286	1.13 ± 0.22	1.10 ± 0.20	0.207
LDL (mmol/L)	2.99 ± 0.67	2.79 ± 0.63	0.126	2.80 ± 1.01	2.87 ± 0.83	0.343
AST, U/L	25.33 ± 14.12	25.67 ± 10.10	0.21	24.78 ± 10.88	21.58 ± 6.26	0.016
ALT, U/L	37.60 ± 27.25	37.66 ± 14.57	0.840	36.81 ± 22.72	31.06 ± 16.36	0.028

BMI, body mass index; PBF, percentage body fat; FBG, fasting blood glucose; HbA1c, glycated hemoglobin A 1c; TC, total cholesterol; TG, triglyceride; HDL, high density lipoprotein; LDL, low density lipoprotein; AST, aspartate aminotransferase; ALT, alanine aminotransferase.

### Effects of KF Treatment on the Diversity and Richness of the Gut Microbiota

**Figure 2:**
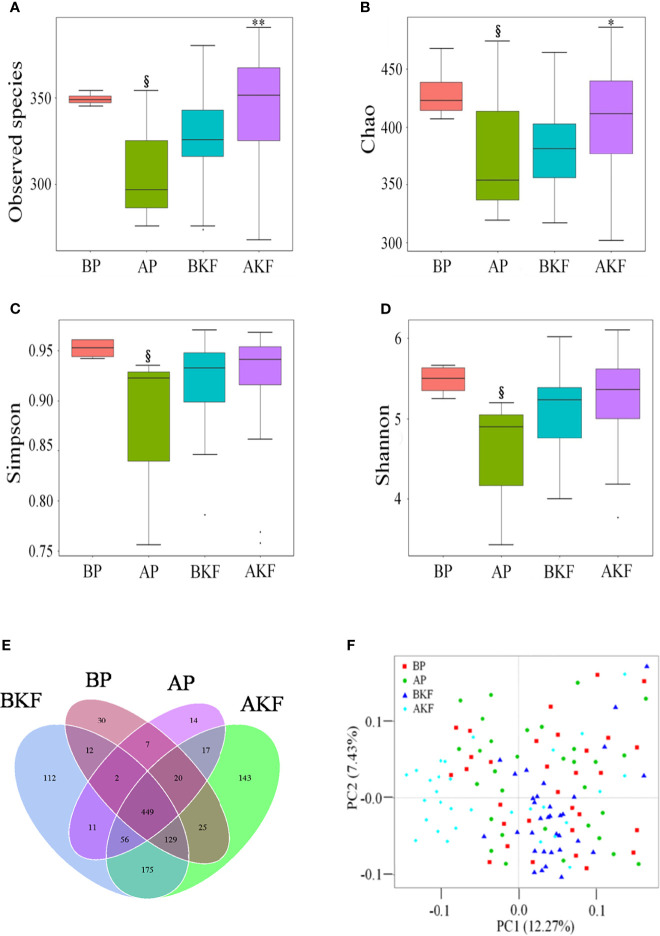
KF treatment on gut microbiota richness and diversity. **(A–D)** α-diversity of the intestinal microbiota was measured by observed species **(A)**, Chao 1 index **(B)**, inverse Simpson index **(C)**, and Shannon index **(D)**. Venn diagram illustrated overlap of OTUs in the gut microbiota among the samples **(E)**. **(F)** β-diversity demonstrated that samples after treatment tender to cluster together and away from the samples before treatment. ^§^ *p <*0.05 versus BP and *p <0.05, ** *p <*0.01 versus BKF. BP, before placebo; AP, after placebo; BKF, before konjaku flour; AKF, after konjaku flour.

Gut microbial alpha diversity metrics, which take into consideration of both richness and evenness, as indicated by the observed species, Chao1 index, Simpson index, and Shannon index. The observed species and Chao1 index significantly increased in the AKF group compared to the BKF group (*p* = 0.0235 and *p* = 0.0044) (**Figures 2A, B**). However, the observed species, Chao1 index, Simpson index, and Shannon index significantly decreased in the AP compared to the BP group (*p* = 0.0104, *p* = 0.0102, *p* = 0.0317, and *p* = 0.0274) (**Figures 2A–D**). These results suggested that KF treatment significantly increased gut microbial alpha diversity.

In order to better understand the richness shared between each group, a Venn diagram showing the overlap between the groups was developed. The analysis showed that only 449 of the total richness of 1,743 OTUs were shared among all samples (**Figure 2E**). The BP group had 674 OTUs, the AP group had 576 OTUs, the BKF group had 946 OTUs, and the AKF group had 1,014 OTUs (**Figure 2E**). In order to measure the degree of similarity between microbial communities, a principal coordinate analysis was used to calculate β diversity (**Figure 2F**). The principal coordinate analysis demonstrated that samples after KF treatment tender to cluster together and away from the samples before treatment. These data demonstrated that KF treatment remarkably improved richness and diversity of intestinal microbiota.

### Effects of KF Treatment on the Gut Microbiota Composition

Gut microbiota composition significantly changed before and after treatment with KF.

**Figure 3:**
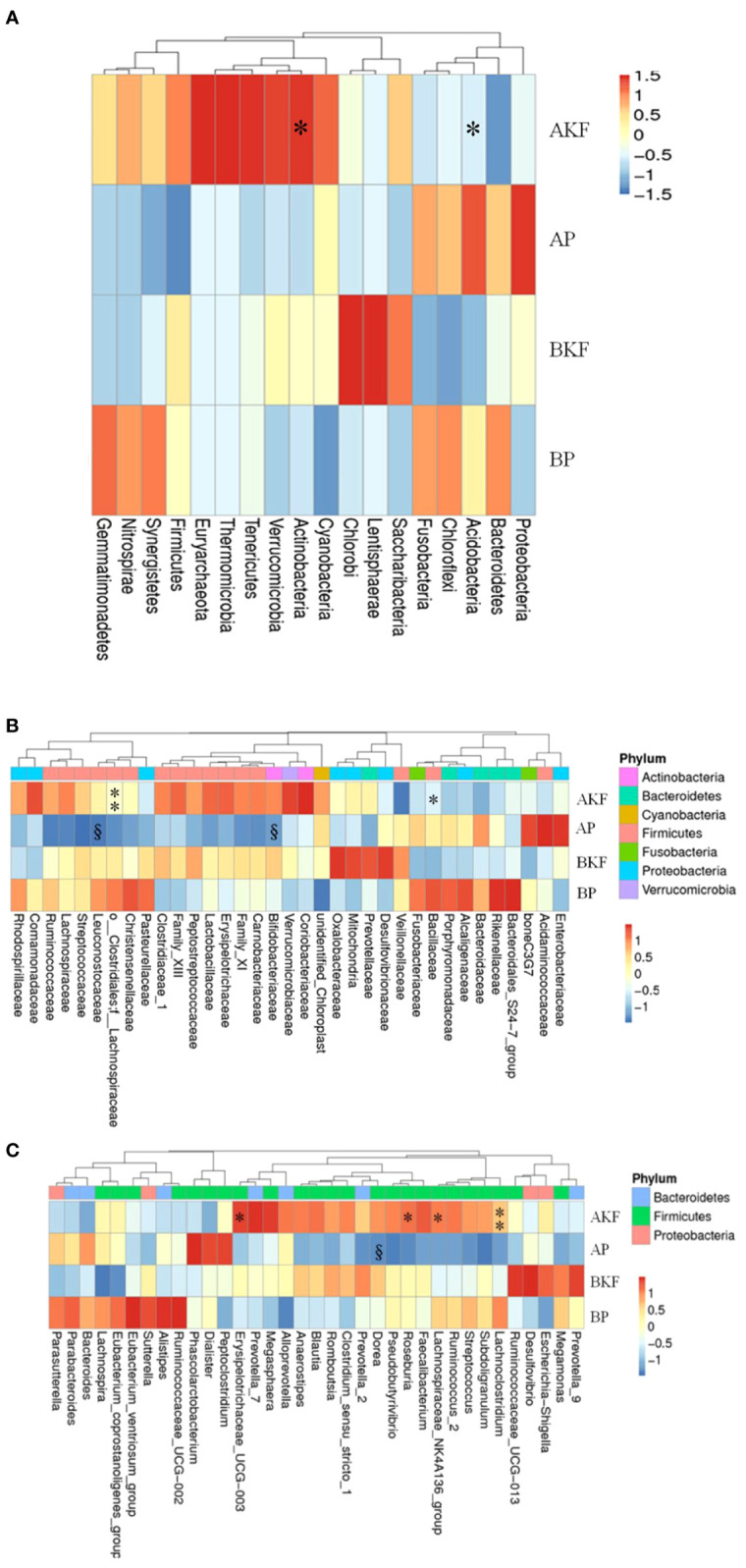
The phyla, family, and genera are significantly different in different groups. **(A)** The relative abundance of the first 18 different phyla; **(B)** The relative abundance of the first 35 different families; **(C)** The relative abundance of the first 35 different genera. By subtracting the average abundance and dividing by the standard deviation of all samples, the abundance profile is converted to a Z-score. When the row abundance is lower than the average, the Z score is negative (shown in blue). ^§^P <0.05 with BP and *p <0.05, **p <0.01 with BKF.

At the phylum level, the relative abundance of *Actinobacteria* and *Acidobacteria* were significantly increased in the AKF group compared to the BKF group (*p <*0.05) (**Figure 3A**).

At the family level, the relative abundance of *Lachnospiraceae*, *Bacillaceae*, *Aerococcaceae*, *Solirubrobacteraceae*, 288-2 and RB41 significantly elevated after treatment with KF, while there was relative abundance of *Sporolactobacillaceae* significantly reduced after treatment with KF (*p <*0.05) (**Figure 3B** and **Table S1**). Furthermore, placebo treatment reduced the relative abundance of *Bifidobacteriaceae* and *Leuconostocaceae* in the AP group compared to the BP group (*p <*0.05) (**Figure 3B** and **Table S1**).

At the genus level, the relative abundance of Roseburia, Lachnoclostridium, Erysipelotrichaceae UCG-003, Lachnospiraceae NK4A136 group, Lachnospiraceae UCG-004, Fusicatenibacter, Lachnospiraceae UCG-003, Clostridium sensu stricto 13, Howardella, Intestinimonas, Bacillus, Holdemania, Epulopiscium, Candidatus, Soleaferrea, Solobacterium, Solirubrobacter, and Abiotrophia significantly increased after KF treatment, while Lactococcus, Eubacterium brachy group, Sporolactobacillus, Geobacillus, and Silanimonas significantly reduced (**Figure 3C** and **Table S1**). In addition, the relative of abundance of Dorea significantly reduced in the AP group compared to BP group (p <0.05) (**Figure 3C** and **Table S1**).

**Figure 4:**
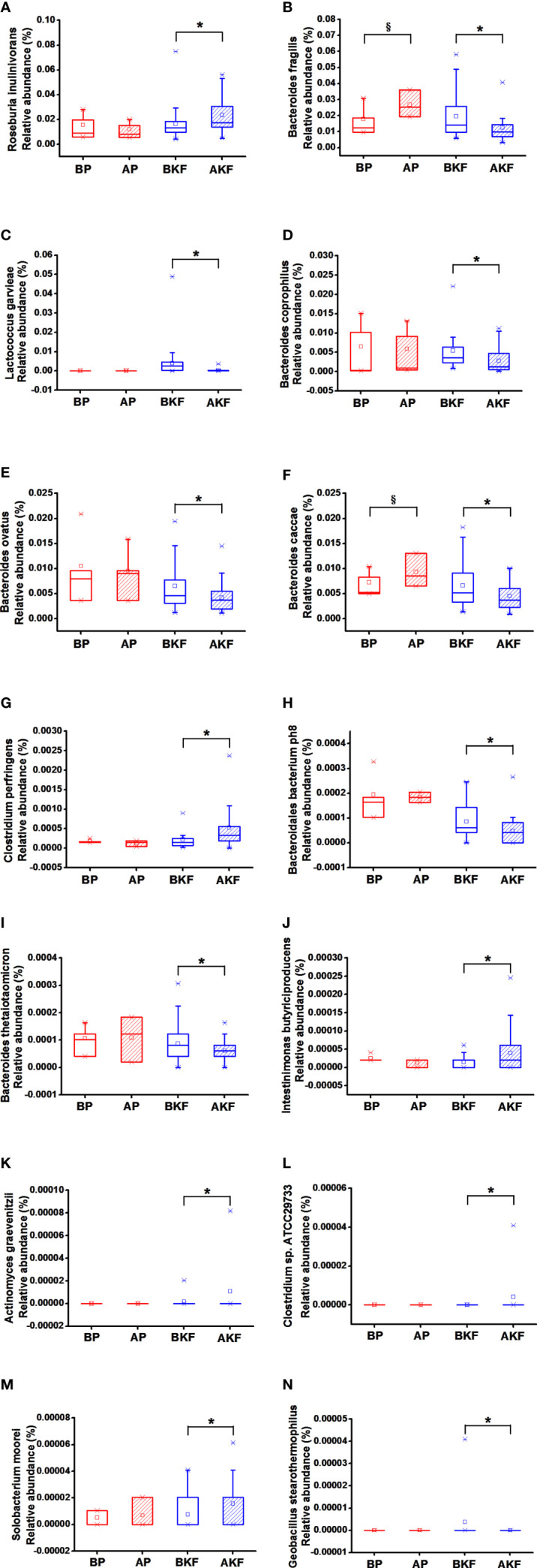
Relative abundance at species levels across groups. **(A)** Roseburia inulinivorans, **(B)** Bacteroides fragilis, **(C)** Lactococcus garvieae, **(D)** Bacteroides coprophilus, **(E)** Bacteroides ovatus, **(F)** Bacteroides caccae, **(G)** Clostridium perfringens, **(H)** Bacteroidales bacterium ph8, **(I)** Bacteroides thetaiotaomicron, **(J)** Intestinimonas butyriciproducens, **(K)** Actinomyces graevenitzii, **(L)** Clostridium sp. ATCC 29733, **(M)** Solobacterium moorei, **(N)** Geobacillus stearothermophilus. ^§^p < 0.05 versus BP and *p <0.05, versus BKF.

At the species level, the relative abundance of Roseburia inulinivorans, Clostridium perfringens, Intestinimonas butyriciproducens, Actinomyces graevenitzii, Clostridium sp. ATCC 29733, and Solobacterium moorei significantly elevated in the AKF group compared to the BKF group, while the relative abundance of the Bacteroides fragilis, Lactococcus garvieae, Bacteroides coprophilus, Bacteroides ovatus, Bacteroides caccae, Bacteroidales bacterium ph8, Bacteroides thetaiotaomicron, and Geobacillus stearothermophilus significantly reduced in the AKF group compared to the BKF group (p <0.05) (**Figures 4A–N**). Moreover, the relative abundance of B. fragilis and B. caccae significantly increased in the AP group compared to BP group (p <0.05) (**Figures 4A–N**).

Altogether, KF treatment can increase some beneficial bacteria and decrease some harmful bacteria.

### Gut Microbiota Associated With Obesity Index, Blood Parameters

**Figure 5:**
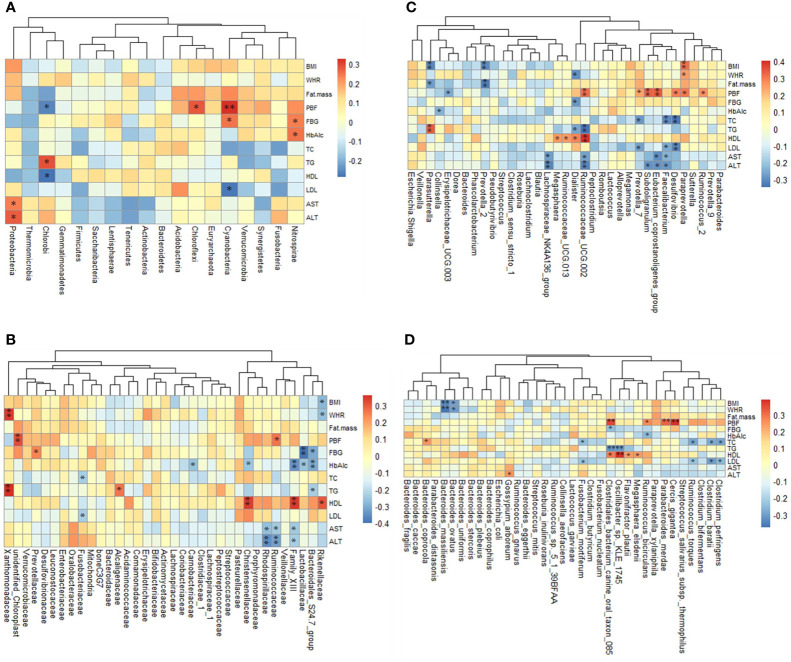
The relationship between obesity indexes or/and blood parameters and taxon across groups is estimated by Spearman’s correlation analysis. **(A)** The 18 top altered phylum, **(B)** the 35 top family, **(C)** the 35 top genera, **(D)** the 35 top genera species. *p <0.05 and **p <0.01.

At the phylum level, five phyla were significantly correlated with obesity index and blood parameters (*p <*0.05 or *p <*0.01) (**Figure 5A**). *Proteobacteria* was related to obesity. For blood parameters, *Proteobacteria* was significantly and positively correlated with serum AST and ALT content (*p <*0.01).

At the family level, thirteen families were significantly correlated with obesity index and blood parameters (*p <*0.05 or *p <*0.01) (**Figure 5B**). *Prevotellaceae* was related to obesity. For blood parameters, *Prevotellaceae* was significantly and positively correlated with serum FBG content (*p <*0.01).

At the genus level, sixteen genera were significantly correlated with obesity index and blood parameters (*p <*0.05 or *p <*0.01) (**Figure 5C**). *Erysipelotrichaceae* UCG.003 and *Lachnospiraceae* NK4A136 were related to KF treatment. For blood parameters, *Erysipelotrichaceae* UCG.003 was significantly and negatively correlated with serum FBG content (*p <*0.01). *Lachnospiraceae* NK4A136 group was significantly and negatively correlated with serum ALT content (*p <*0.01).

At the species level, fifteen species significantly correlated with obesity index and blood parameters (*p <*0.05 or *p <*0.01) (**Figure 5D**). *Bacteroides ovatus* were related to KF treatment. For obesity index, *B. ovatus* abundance was significantly and negatively correlated with BMI and WHR (*p <*0.01). For blood parameters, *C. perfringens* were significantly and negatively correlated with serum TC and LDL content (*p <*0.01).

Altogether, these bacteria can influence obesity index and blood parameters.

## Discussion

Obesity is a multi-factor problem, and many related factors need to be considered when designing interventions that may be successful. Recent literature provides evidence that the gut microbiota may be related to the cause of obesity. In this regard, we conducted this study to evaluate the effect of KF consumption on the body weight and fat mass of obese subjects. The consumption of KF led to some beneficial changes in the health indicators of overweight and obese subjects.

KF consumption impacts on obesity indexes and blood parameters. BMI, fat mass, serum PBF, HbA1c, TG, AST, and ALT significantly decreased in KF consumers (*p <*0.05 or *p <*0.01).

The gut microbiome has a major impact on obesity, metabolic syndrome, liver steatosis, and the metabolic balance of the host immune system (Zhang et al., 2017). Intestinal flora imbalance is related to obesity and metabolic disorders (Lynch and Pedersen, 2016). KF treatment significantly increased α diversity of the intestinal microbiota in obese subjects. Reduced diversity of the gut microbiota is associated with obesity, systemic inflammation, and metabolic diseases. In contrast, increased microbial diversity is associated with improved metabolic health (Sonnenburg and Backhed, 2016).

KF treatment also altered the structure of gut microbiota. At the phylum level, the abundance of *Actinobacteria* and *Acidobacteria* were significantly increased in KF consumers (*p <*0.01). Clarke et al. (2013) found that treatment of diet-induced obese mice resulted in significant increase in the relative proportion of *Actinobacteria*. At the family level, the relative abundance of *Lachnospiraceae*, *Bacillaceae*, *Aerococcaceae*, *Solirubrobacteraceae*, 288-2 and RB41 remarkably elevated in KF consumers, while the relative abundance of the *Sporolactobacillaceae* significantly reduced in KF consumers (p <0.05). Several studies suggested that the relative abundance of *Lachnospiraceae* significantly decreased in patients with cirrhosis and fatty liver (Chen et al., 2011; Bajaj et al., 2012; Zhu et al., 2013). Moreover, Clarke et al. (2013) found *Lachnospiraceae* was negatively correlated with body weight.

At the genus level, the relative abundance of Roseburia, Lachnoclostridium, Erysipelotrichaceae UCG-003, Lachnospiraceae NK4A136 group, Lachnospiraceae UCG-004, Fusicatenibacter, Lachnospiraceae UCG-003, Clostridium sensu stricto 13, Howardella, Intestinimonas, Bacillus, Holdemania, Epulopiscium, Candidatus, Soleaferrea, Solobacterium, Solirubrobacter, and Abiotrophia significantly increased in KF consumers (p <0.05, p <0.01 or p <0.001), while the relative abundance of Lactococcus, Eubacterium brachy group, Sporolactobacillus, Geobacillus, and Silanimonas significantly reduced in KF consumers (p <0.05). Some studies demonstrated that the relative abundance of Roseburia significantly reduced in obese mice (Dewulf et al., 2011; Neyrinck et al., 2011). Neyrinck et al. (2011) found the increase of Roseburia and also the decrease of body weight and fat mass were in diet-induced obese mice treated with prebiotic wheat arabinoxylan. Roseburia is one of the main producers of butyrate. Butyrate can regulate the function and migration of neutrophils, inhibit the expression of vascular cell adhesion molecule-1 induced by inflammatory cytokines, increase the expression of tight junction proteins in colonic epithelial cells, and reduce the release of cells from human immune cells factors and chemokines to exert anti-inflammatory effects (Nicholson et al., 2012). Therefore, n-butyrate or specific types of butyrate-producing gut bacteria may be a new target for restoring host immune function and barrier integrity and regulating energy metabolism (Nicholson et al., 2012). n-Butyrate can also be used directly by colonic epithelial cells to produce ketone bodies and carbon dioxide (Nicholson et al., 2012). Andoh et al. (2016) demonstrated the relative abundance of Solobacterium reduced significantly in obese patients. Some studies also found Lactococcus significantly increased in diet-induced obese mice. Furthermore, treatment of diet-induced obese mice resulted in the significant increase of Roseburia and the marked decrease of body weight and fat mass (Ravussin et al., 2012; Jung et al., 2016).

At the species level, the relative abundance of *R. inulinivorans*, *C. perfringens*, *I. butyriciproducens*, *A. graevenitzii*, *C.* sp. ATCC 29733, and *S. moorei* significantly elevated in KF consumers, while the relative abundance of the *B. fragilis*, *L. garvieae*, *B. coprophilus*, *B. ovatus*, *B. caccae*, *B. bacterium* ph8, *B. thetaiotaomicron*, and *G. stearothermophilus* significantly reduced in KF consumers (*p <*0.05). *R. inulinivorans* and *I. butyriciproducens* are the main producers of butyrate, and they are potential probiotics (Flint et al., 2007; Kläring et al., 2013). Zuo et al. (2011) found that the relative abundance of *C. perfringens* markedly reduced in obese participants. Vael et al. (2011) showed that a high concentration of *B. fragilis* in infants between 3 weeks and 1 year old is associated with a higher risk of obesity in the future. Collado et al. (2008) showed that high concentrations of *B. fragilis* are associated with excessive weight gain during pregnancy. *L. garvieae* is one of the pathogenic bacteria (O’Connor et al., 2015). Heo et al. (2016) had shown that probiotics and *Garcinia cambogia* extract alleviated weight gain and adiposity in HFD fed mice, in part *via* differentially modulating the composition of intestinal microbiota, namely, a significant decrease in the relative abundance of *L. garvieae*. Andoh et al. (2016) found that the relative abundance of *B. coprophilus* and *B. ovatus* dramatically increased in obese patients. Dicksved et al. (2008) also demonstrated that subjects with Crohn’s disease had a higher relative abundances of *B. ovatus*. Giongo et al. (2011) found the relative abundance of *B. ovatus* significantly increased in type 1 diabetes (T1D)-associated autoimmune children who are at high genetic risk for this disorder compared to non-autoimmune individuals. It is known that *B. thetaiotaomicron* can decompose many other indigestible dietary plant polysaccharides (Xu et al., 2003). Hooper et al. (2001) found that *B. thetaiotaomicron* could regulate production of ileal epithelial fucosylated glycans for its own nutritional benefit. Furthermore, Armougom et al. (Samuel and Gordon, 2006) found *B. thetaiotaomicron* was related to obesity

Additionally, in all of the significantly changed gut microbiota after KF treatment, *Erysipelotrichaceae* UCG.003, *Lachnospiraceae* NK4A136 group, *B. ovatus* and *C. perfringens* were dramatically correlated with obesity index and blood parameters. *Erysipelotrichaceae* UCG.003 was dramatically and negatively correlated with PBF (*p <*0.01). *Lachnospiraceae* NK4A136 group was significantly and negatively correlated with serum AST and ALT content (*p <*0.01). Altogether, these results suggest that these bacteria play important roles in the development of obesity.

### Conclusion

KF intervention could reduce obesity indexes and blood parameters, regulate the diversity and composition of the gut microbiota, accelerate growth of good bacteria, and inhibit growth of maleficent bacteria. In addition, changes in the microbiota have a strong correlation with obesity index and blood parameters. These findings support that the anti-obesity and other effects of KF may be mediated through its selective regulation of the microbiota. The duration of trial treatment is short, which maybe a limitation of our study. Then we would focus on KF and make further investigation.

## Data Availability Statement

The original contributions presented in the study are included in the article/**Supplementary Material**. Further inquiries can be directed to the corresponding authors.

## Ethics Statement

The studies involving human participants were reviewed and approved by the ethical committee of Medical Faculty, Kunming University of Science and Technology. The patients/participants provided their written informed consent to participate in this study.

## Author Contributions

XK, JH and YK conceived the research project; YL, YD, MC, LG, XH, TL and SC performed the experiments; JH, FYa and FYu collected samples; YD analyzed the data; YL wrote the draft; YK revised the manuscript; XK and JH supervised the work. All authors read and approved the final manuscript.

## Funding

This work was supported by the Science Research Start-up Fund for Doctor of Shanxi Medical University (Grant No. XD1807), the Science Research Start-up Fund for Doctor of Shanxi Province (Grant No. SD1807), the Scientific and Technological Innovation Programs of Higher Education Institutions in Shanxi (Grant No. 2019L0425) and the Shanxi Province Science Foundation for Youths (Grant No. 201901D211314).

## Conflict of Interest

The authors declare that the research was conducted in the absence of any commercial or financial relationships that could be construed as a potential conflict of interest.

## Publisher’s Note

All claims expressed in this article are solely those of the authors and do not necessarily represent those of their affiliated organizations, or those of the publisher, the editors and the reviewers. Any product that may be evaluated in this article, or claim that may be made by its manufacturer, is not guaranteed or endorsed by the publisher.
