# Supplementary material for: Effects of Konjaku Flour on the Gut Microbiota of Obese Patients
Source: Front Cell Infect Microbiol. 2022 Mar 1;12:771748. doi: 10.3389/fcimb.2022.771748 (PMC8921482; doi:10.3389/fcimb.2022.771748)
Supplement: Supplementary file 1 [file Table_1.docx]

Table S1 Relative abundance of family and genera except the top 35 family and genera across groups

| Taxon | | | BP | AP |  |  | BKF | AKF |  |
| --- | --- | --- | --- | --- | --- | --- | --- | --- | --- |
|  |  |  | mean | mean | p value |  | mean | mean | p value |
| Family |  | *Aerococcaceae* | NA | NA | NA |  | 0.000004 | 0.000011 | 0.022 |
|  |  | *Solirubrobacteraceae* | 0.000015 | 0.000012 | 0.058 |  | 0.000001 | 0.000006 | 0.023 |
|  |  | 288-2 | 0.000009 | 0.000007 | 0.508 |  | 0.000000 | 0.000003 | 0.023 |
|  |  | *Sporolactobacillaceae* | 0.000005 | 0.000004 | 0.391 |  | 0.000004 | 0.000000 | 0.017 |
|  |  | RB41 | 0.000002 | 0.000003 | 0.423 |  | 0.000000 | 0.000003 | 0.043 |
| Genus |  | *Lactococcus* | 0.000066 | 0.000054 | 0.489 |  | 0.004024 | 0.000673 | 0.018 |
|  |  | *Lachnospiraceae* UCG-004 | 0.004417 | 0.004809 | 0.218 |  | 0.002685 | 0.003710 | 0.036 |
|  |  | *Fusicatenibacter* | 0.001508 | 0.001452 | 0.107 |  | 0.001986 | 0.002867 | 0.005 |
|  |  | *Lachnospiraceae* UCG-003 | 0.000143 | 0.000114 | 0.165 |  | 0.000095 | 0.000487 | 0.016 |
|  |  | *Clostridium sensu stricto* 13 | NA | NA | NA |  | 0.000002 | 0.000089 | 0.005 |
|  |  | *Howardella* | 0.000046 | 0.000037 | 0.403 |  | 0.000043 | 0.000104 | 0.012 |
|  |  | *Intestinimonas* | 0.000026 | 0.000021 | 0.234 |  | 0.000015 | 0.000040 | 0.018 |
|  |  | *Bacillus* | 0.000102 | 0.000089 | 0.661 |  | 0.000028 | 0.000052 | 0.026 |
|  |  | *Holdemania* | 0.000066 | 0.000068 | 0.686 |  | 0.000038 | 0.000074 | 0.001 |
|  |  | *Epulopiscium* | NA | NA | NA |  | 0.000001 | 0.000009 | 0.038 |
|  |  | *Candidatus Soleaferrea* | 0.000015 | 0.000014 | 0.391 |  | 0.000002 | 0.000010 | 0.048 |
|  |  | *Solobacterium* | 0.000006 | 0.000007 | 0.423 |  | 0.000007 | 0.000016 | 0.030 |
|  |  | *Eubacterium brachy* group | 0.000005 | 0.000004 | 0.391 |  | 0.000006 | 0.000001 | 0.017 |
|  |  | *Solirubrobacter* | 0.000015 | 0.000011 | 0.058 |  | 0.000001 | 0.000006 | 0.023 |
|  |  | *Abiotrophia* | NA | NA | NA |  | 0.000003 | 0.000010 | 0.015 |
|  |  | *Sporolactobacillus* | 0.000005 | 0.000003 | 0.391 |  | 0.000004 | 0.000000 | 0.017 |
|  |  | *Geobacillus* | NA | NA | NA |  | 0.000004 | 0.000000 | 0.032 |
|  |  | *Silanimonas* | NA | NA | NA |  | 0.000002 | 0.000000 | 0.044 |

NA, not applicable.
